# Gastric-type endocervical adenocarcinoma with mucoepithelial metaplasia combined with a serous borderline tumor: A case report

**DOI:** 10.1097/MD.0000000000028239

**Published:** 2021-12-23

**Authors:** Man Yin, Linqing Yang, Yunfei Wang

**Affiliations:** a Department of Clinical Medicine, Jining Medical University, Jining, Shandong, China; b Affiliated Hospital of Jining Medical University, Jining, Shandong, China.

**Keywords:** adenocarcinoma, cervical cancer, gastric type

## Abstract

**Rationale:**

Gastric-type endocervical adenocarcinoma (GAS) is a rare type of cervical adenocarcinoma that is a mucinous adenocarcinoma with a variety of gastral patterns. To date, there are no systematic clinical diagnosis and treatment guidelines.

**Patient concerns:**

In our case, a 49-year-old woman underwent pelvic magnetic resonance imaging (MRI) due to a pelvic mass, and cervical lesions were unexpectedly found. After receiving relevant surgical treatment, the pathological results showed the particularity of the tumor type—cervical gastric adenocarcinoma with a borderline serous tumor of both appendages and the right ovary.

**Diagnoses:**

Postoperative routine pathological examination showed mucoepithelial metaplasia accompanied by a borderline serous tumor.

**Interventions:**

After gynecological/urinary ultrasound, blood tests, MRI, cervical biopsy, and uterine curettage, “robot-assisted laparoscopic radical hysterectomy + bilateral salpingectomy-ovariectomy + pelvic lymph node dissection + pelvic adhesiolysis” were performed. After the surgery, the patient was treated with radiotherapy and concurrent chemotherapy.

**Outcomes:**

After the operation, radiotherapy, and chemotherapy, the patient had no tumor recurrence and is still in good condition.

**Lessons:**

The diagnosis of GAS is relatively difficult, its clinical manifestations lack specificity, and the pathogenesis has nothing to do with human papillomavirus infection. The patient was misdiagnosed with vaginitis at a local hospital. However, we found that MRI and pathological examination were helpful for the diagnosis of the disease. Although there are no relevant guidelines to explain the treatment principles of GAS, we believe that early surgery is conducive to the prognosis of the disease because GAS has a certain tolerance to radiotherapy and chemotherapy.

## 1 Introduction

Although cervical cancer is the only cancer that can be cured and prevented, it is still the fourth most common cancer among women in the world. The incidence rate of cervical cancer in women is high. In 2018, more than 500,000 new cases of cervical cancer were reported worldwide, and 310,000 women died from cervical cancer.^[1,2]^ Human papillomavirus (HPV) infection is the main cause of cervical cancer.^[3,4]^ However, gastric-type endocervical adenocarcinoma (GAS) was first proposed in 2007.^[5]^ The incidence of GAS has nothing to do with HPV, and its clinical manifestations are not specific, which poses a challenge to the early diagnosis and treatment of cervical cancer. According to a literature search in PubMed, we found 97 studies related to GAS, but there were no reports of comorbid borderline serous tumors. In this patient, we found a mucoepithelial metaplasia GAS along with a serous borderline tumor, which was diagnosed according to histopathology and immunohistochemistry. This patient was treated for the first time at the Department of Gynecology, Affiliated Hospital of Jining Medical University. The patient provided signed informed consent.

## 2 Case report

**Figure 1:**
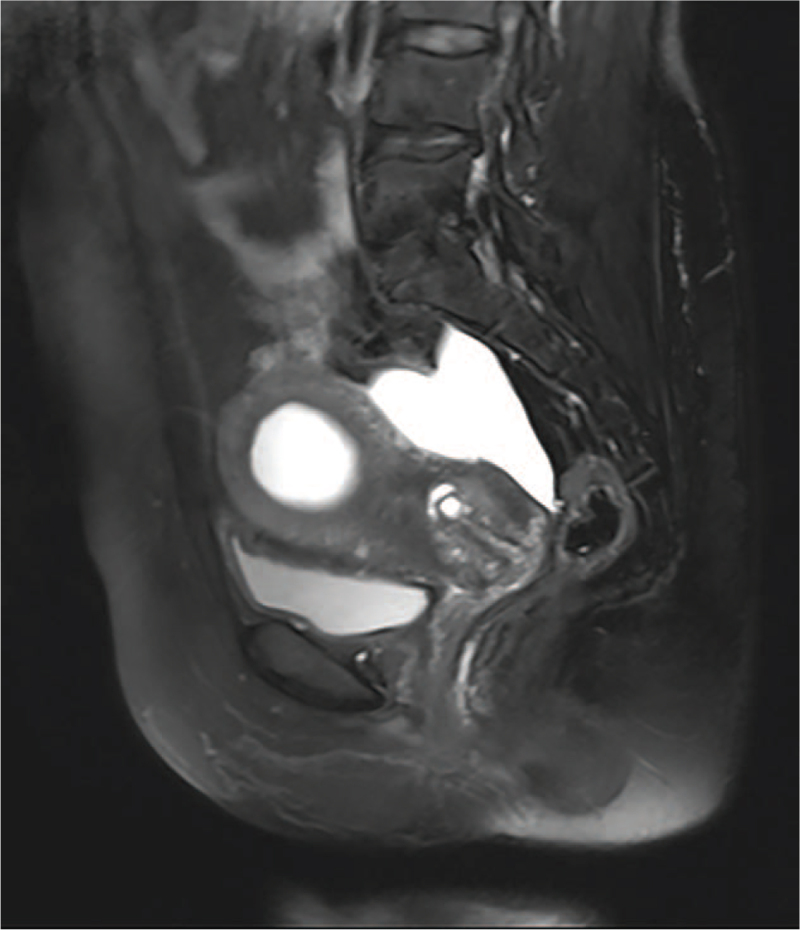
Thickening of the posterior wall of the cervix with a mass-like signal shadow suggested by MRI. MRI = magnetic resonance imaging.

**Figure 2:**
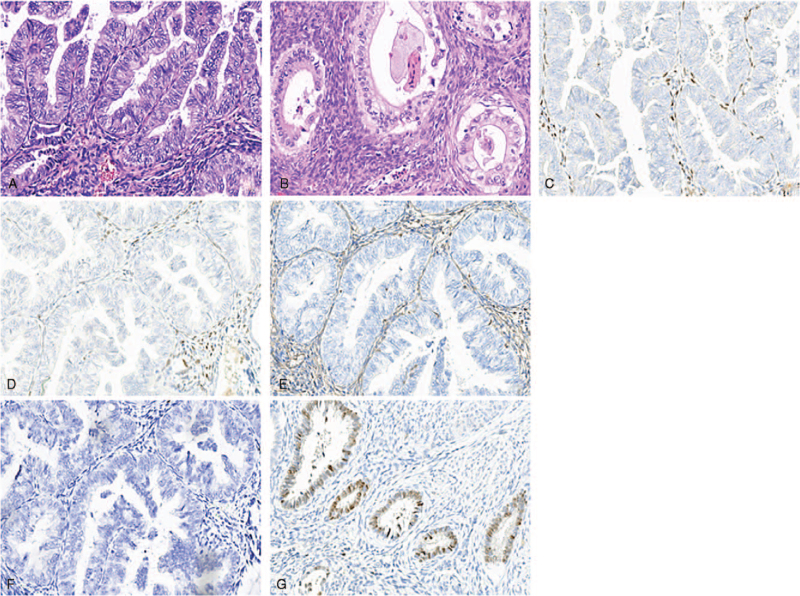
The results of HE staining (2A–2B) and immunohistochemical examination (2C–2G) viewed with a microscope. (2A) Gastric-type adenocarcinoma of the cervix with mucoepithelial metaplasia (HE ×200); (2B) Borderline clear cell tumor of the right ovary (HE ×200); (2C) ER (–) of the cervix (×200); (2D) PR (–) of the cervix (×200); (2E) Vimentin (–) of the cervix (×200); (2F) P16 (–) of the cervix (×200); (2G) P53 (+) of the cervix.

A 49-year-old patient was admitted to the hospital due to “the discovery of uterine cavity space-occupying and pelvic mass for over 20 days” on August 30, 2020. The patient had regular menstruation at normal times, with the chief complaint of increased vaginal discharge (transparent in color with no odor) with the same volume as that of menstruation over the last 10 years. The patient underwent “hysteroscopic resection of the uterine cavity lesion” 3 years ago due to a “uterine cavity space-occupying mass”, with “adenomyoma” indicated by postoperative pathology, and the patient showed improvement in vaginal discharge symptoms postoperatively. In the last year, the patient experienced symptoms of increased vaginal discharge again with no obvious inducement. The discharge volume was approximately twice that of the normal menstrual volume, and there was occasionally blood in leucorrhea, without odor, accompanied by abdominal distension; no abdominal pain, frequent urination, urgent urination, dysuria, fever, nausea, vomiting, or other discomfort were reported. The patient visited the local hospital many times, received a diagnosis of vaginitis and was provided with drug therapy with poor effects. On August 10, 2020, the patient underwent color Doppler ultrasound in the local hospital with the discovery of an “abnormal echo in the uterine cavity (3.9 cm × 3.4 cm × 1.6 cm) and cystic mass in bilateral adnexa areas”. The local hospital recommended a further examination, and the patient refused due to personal reasons. For further diagnosis and treatment, the patient visited our hospital on August 30, 2020, and was admitted to our hospital due to an “abnormal echo in the uterine cavity, pelvic infection, adnexal tumor, and hydrosalpinx?”. The personal history and family history of the patient were unremarkable. Gynecological examination results were as follows: No abnormality in the development of the vulva; no obstruction in the vagina; an increase in vaginal discharge, which was odorless; cervical congestion; bleeding on touch; tough texture; large size of the uterine body, with normal activity and tenderness pain; and tenderness pain in the bilateral appendages. On July 15, 2020, the detection result of HPV-DNA typing in the cervix was negative; however, there were abnormalities in the ThinPrep Cytology Test. Re-examination by transvaginal ultrasound on August 30, 2020, suggested a mixed-echo mass in the uterine cavity, a bilateral adnexal cystic mass, a left adnexal cystic mass, an isoechoic mass in the right adnexal area (with the possibility of teratoma to be confirmed), multiple cystic nodules of the cervix, and pelvic effusion. Tumor marker detection showed no abnormalities in human epididymis protein 4, carbohydrate antigen 125, the premenopausal ROMA index, carcinoembryonic antigen, alpha fetoprotein, or specific β human chorionic gonadotropin. Furthermore, carbohydrate antigen 199 was measured to be 170.43 U/mL (reference range: 0–37 U/mL). Laparoscopic hysteroscopy was planned. However, considering the presence of a space-occupying lesion in the uterine cavity, a pelvic magnetic resonance imaging (MRI) plain scan and diffusion-weighted imaging were performed on August 31, 2020 (Fig. 1). As indicated by the results, the posterior wall of the cervix was thickened, with a mass-like shadow (approximately 2.8 cm × 1.1 cm × 1.4 cm in size) of slightly low signal intensity on T1WI and slightly high signal intensity on T2WI, with unclear boundaries and an uneven distribution of signals; in addition, diffusion was limited slightly in DWI and showed a high signal (no further description of the remaining imaging results). The imaging diagnosis was as follows: abnormal signal of the cervical posterior wall, with the consideration of a neoplastic lesion and the possibility of cervical cancer; abnormal signal nodule in the right adnexal area, with the consideration of the possibility of tumor or a chocolate cyst, which remain to be differentiated; an intrauterine mass-like abnormal signal shadow with a close relationship with the cervix locally, with the consideration of the possibility of cervical lesions (polyps or endometrial hyperplasia) protruding into the uterine cavity; bilateral hydrosalpinx and dilation and complicated hematocele in the right fallopian tube; cervical myometrium signal disorder, multiple cysts, and a bleeding focus, with the possibility of endometriosis to be determined; and a small amount of pelvic effusion. Consequently, the patient underwent cervical colposcopy + cervical biopsy + uterine curettage. On September 5, 2020, pathological examination findings based on cervical biopsy + uterine curettage suggested the following: (uterine cavity) proliferative endometrium accompanied by a few papillary structures; (cervical canal) the detection of abnormal glands with a papillary structure in the bleeding tissues, immunohistochemistry: P53 (+, 20%–30%), Ki-67 (+, 30%–40% partially), Vimentin (–), ER (–), PR (–), and P16 (–) in the heterotypic gland; and (at 3, 6, 9, and 12 o’clock in the cervix) heterotypic glands in the tissues tested, and adenocarcinoma in situ at least observed by immunohistochemistry, immunohistochemistry: P16 (–), and Ki-67 (+, 60%–70% partially). The pathological examination results showed cervical cancer, which was at stage IB2 on the basis of pre-operative clinical diagnosis. After full informed consent was obtained, “robot-assisted laparoscopic radical hysterectomy + bilateral salpingectomy-ovariectomy + pelvic lymph node dissection + pelvic adhesiolysis” were performed on September 6, 2020. The intra-operative rapid pathological examination showed that the cervical canal was approximately 3 cm in length, with a diameter of the opening of 2.5 cm and severe erosion. At the location of the incision, an invasive tumor was found, which was approximately 3 × 2 × 1.5 cm in size, showing a grayish white cutting section, hard texture, and unclear boundary. The following were suggested by the results: (left side) cystic dilatation with atypical epithelial hyperplasia in the fallopian tube; (right ovary) atypical hyperplastic glands in the stroma with the specific type to be determined by paraffin-embedded section examination; and (cervix) adenocarcinoma. On September 13, 2020, postoperative routine pathological examination findings (Fig. 2) suggested the following: (cervix) invasive gastric adenocarcinoma with a borderline serous tumor, a mass size of 3 × 2 × 1.5 cm, the mass invaded over 1/2 of the cervical wall, and multiple-focal mucous metaplasia; borderline serous tumor of bilateral fallopian tubes; borderline clear cell tumor of the right ovary, with the size of 2.5 × 2 cm; no cancer at the cutting edge of the vaginal wall; proliferative endometrium; and (left pelvic cavity, right pelvic cavity, adjacent area of left common iliac artery, and adjacent area of right common iliac artery) no cancer in the lymph nodes, which were (0/8; 0/8; 0/1; 0/9), respectively. The immunohistochemistry results were as follows: gastric adenocarcinoma, ER, PR, P16, and Vim (–), with P53 (+, positive cell count of 50%–60%); borderline serous tumor, ER, PR, P16, Vim, and WT-1 (–), P53 (+, positive cell count of 3%–5%); and borderline clear cell tumor, ER, PR, P16 (–), P504S (+), and NapsinA (+ partially). One month after the operation, the patient came and received local radiotherapy (DT = 48.6 Gy/27f) in the Department of Oncology of our hospital. During radiotherapy, the patient was given concurrent chemotherapy by using cisplatin (40 mg). After radiotherapy and chemotherapy, the patient was in good condition. No obvious abnormality was found in the test results, and no recurrence was observed. The patient is currently under further follow-up.

## 3 Discussion and conclusion

GAS was proposed by Japanese scholar Kojima et al^[5]^ in 2007 for the first time; GAS was described as a malignant epithelial tumor of the cervix characterized by the expression of gastric mucus and was morphologically similar to adenocarcinoma of the pyloric gland epithelium. In 2014, the WHO classified GAS as a special type of cervical mucinous adenocarcinoma.^[6]^ The clinical symptoms of GAS lack specificity, and it is difficult to diagnose in the early stage, but it has high invasion and rapid development in terms of its biological behaviors, indicating a poor prognosis.

To date, the incidence rate and global distribution of GAS are not known; there are conflicting reports about the proportion of GAS among cervical adenocarcinomas, with 10% in international research^[7]^ and 25% to 28% in Japan.^[5,8]^ The age of patients with GAS ranges from 37 to 84 years old, with a mean age of 49 years old. In terms of clinical symptoms, GAS lacks specificity and manifests primarily as vaginal discharge or irregular bleeding and thin colorless leucorrhea with no odor; therefore, GAS is prone to be misdiagnosed as “vaginitis”. In clinical examination, patients with GAS may show cervical hypertrophy, the so-called “barrel-shaped” cervix; however, in some patients GAS may display no abnormality in appearance during colposcopy. Compared with UEA, GAS is more invasive and usually manifests as deep stromal infiltration of the tumor, lymphatic vessel invasion, lymph node metastasis, ovarian involvement, other pelvic organ involvement, peritoneal dissemination, etc.^[8]^ The pathogenesis of GAS is still unclear. In the past 20 years, a number of studies have found mutations in the serine/threonine kinase 11/liver kinase B1 (STK11/LKB1) gene in the lineage lesions from lobular endocervical glandular hyperplasia and minimal deviation adenocarcinoma of the cervix (MDA) to GAS. This gene is recognized to be a tumor suppressor gene, and its mutation can lead to an incomplete catalytic domain and the loss of kinase activity. At present, STK11/LKB1 is considered to be involved in cell proliferation, cell polarity, cell migration, the DNA damage response, and cell differentiation by regulating the mTOR/AMPK, TGFβ, and/or Wnt signaling pathways. Approximately 50% of sporadic MDA patients have STK11/LKB1 mutations, which are also associated with poor prognosis.^[9]^ However, it should be noted that there is a complex mechanism of STK11/LKB1 mutation in tumorigenesis, which deserves further study.

Considering the absence of specific symptoms and clinical examination findings in GAS, it is difficult to diagnose with ultrasound, computed tomography, and other imaging techniques. In our patient, transvaginal ultrasound examination showed the existence of multiple cystic nodules in the cervix only, which was not helpful for the diagnosis of GAS. As evidenced by the report of Saida et al,^[10]^ MRI was the best choice to show the characteristics of GAS among ultrasound, computed tomography, and MRI. Furthermore, relevant studies^[10,11]^ indicated that MRI exhibited superiority in reflecting the pathological results of some adenocarcinomas and was the preferred imaging method. In MRI examination of GAS, T1WI may show low signal intensity, and T2WI shows high signal intensity; in addition, there may be multiple solid-signal shadows of mixed cystic components in the cervix, and the cysts may be obviously enhanced after enhancement. In this patient, pelvic MRI showed a thickened posterior wall of the cervix, a mass-like low signal on T1WI and a slightly high signal on T2WI, with the possibility of cervical cancer being identified. These findings suggest that MRI helps to diagnose GAS and avoids missed diagnoses and is therefore worth recommending.

Kojima et al^[5]^ established 3 morphological diagnostic criteria for GAS in 2007: an abundant mucinous cytoplasm; a transparent or slightly eosinophilic cytoplasm; and a clear cell boundary. Furthermore, in 2019, Pirog et al^[12]^ extended the morphological description of GAS to glands composed of small cubic cells or flat cells, papillary glands, goblet cells mixed in the gland, and a thick eosinophilic or foamy cytoplasm. By definition, GAS is a group of tumors with great morphological heterogeneity. Typical MDA has only focal atypia, which is not obvious; visible or non-obvious nucleoli; and occasionally visible mitosis. However, there is serious cytological atypia of GAS with moderate and low differentiation. The case we reported was accompanied by mucoepithelial metaplasia and a borderline serous tumor, which is very rare in the clinical setting. It has been reported^[13]^ that the simultaneous occurrence of mucoepithelial metaplasia and tumors in the female genital tract exhibited an intimate association with gastric-type mucinous lesions, and there might be a certain relationship with GAS; however, the relationship still needs to be further clarified, which would contribute to the early diagnosis of GAS. In addition, the patient in our case report had bilateral borderline serous tumors of the fallopian tubes and clear cell tumors of the right ovary, both of which were accompanied by tumor-like changes at the junction of bilateral adnexa. Nevertheless, no similar report has been published thus far.

Immunohistochemistry can be an auxiliary tool for the diagnosis of GAS considering its confusing cellular histological features and high risk of missed diagnosis. Immunohistochemically, GAS is generally characterized as follows: negative ER and PR; positive MUC6 and HIK1083; CK7, P53, and carcinoembryonic antigen expression; the Ki-67 proliferation index is higher than that of normal cervical glands; and P16 is usually negative or focally positive since GAS is considered to have no relationship with HPV infection.^[14]^ The mucus secreted by GAS tumor cells is mainly composed of neutral cytoplasmic mucin. The cervical glands of GAS are generally stained red, while those of a normal cervix are purple when stained with alcian blue-periodic acid Schiff.^[15]^ The patient we tested showed negative ER, PR and P16, and positive P53, and the Ki-67 proliferation index of the cervical glands of GAS was 60% to 70% higher than that of normal cervical glands. Clearly, the aforementioned results are basically consistent with those reported in the relevant literature and may be conducive to further clarification of GAS diagnosis.

To date, there is no available treatment principle for GAS based on its histopathological types and molecular pathological characteristics. As recommended by the 2015 National Comprehensive Cancer Network Clinical Practice Guidelines in Oncology for Uterine Neoplasms, the treatment for patients with GAS is similar to that of patients with squamous cell carcinoma at the same stage. In other words, radical hysterectomy is recommended for patients with stage IB1 and stage IIA1 disease, while for patients with stage IB2, IIA2, and IIB–IVa disease, the recommended scheme is platinum-based concurrent chemoradiotherapy.^[16]^ Furthermore, despite an update to the 2019 National Comprehensive Cancer Network guidelines concerning the treatment principles of common cervical adenocarcinoma according to its histopathological classification, it suggests only that fertility-preserving surgery should not be performed for treating patients with GAS.^[17]^ Moreover, it has been reported that GAS has a certain tolerance to radiotherapy and chemotherapy.^[18]^ Consequently, a consensus has been reached in the clinical setting that early diagnosis, clinical stage, and surgical methods significantly affect the prognosis of patients. GAS has an extremely poor prognostic outcome, with a 5-year disease-free survival rate of only 30%.^[19]^ Delayed diagnosis due to HPV negativity and difficulty screening is the major factor contributing to the poor prognosis of GAS. Accordingly, early discovery, early diagnosis, and early treatment, as occurred for this patient, will substantially contribute to improvements in the prognosis and survival rate of patients with GAS.

In summary, HPV negativity is one of the main causes of the delayed diagnosis of GAS. However, there are few studies on the pathogenesis and treatment of non-HPV-associated GAS; thus, clinicians face enormous challenges in the diagnosis and treatment of GAS. The significance of this case report lies in the fact that according to the patient's clinical manifestations, a clear diagnosis was possible with the combination of MRI, pathology, immunohistochemistry, and so on. Therefore, it is hoped that the combination of these methods to achieve early detection and early diagnosis as much as possible will eventually facilitate the selection of appropriate treatment to maximize the benefits to patients.

## **A**cknowledgments

The author thanks our hospital colleagues for their support and the patient's dedication.

## Author contributions

**Conceptualization:** Yunfei Wang.

**Data curation:** Man Yin, Yunfei Wang.

**Methodology:** Linqing Yang, Yunfei Wang.

**Writing – original draft:** Man Yin.

**Writing – review & editing:** Linqing Yang, Yunfei Wang.
